# The cadmium oxidotellurates(IV) Cd_5_(TeO_3_)_4_(NO_3_)_2_ and Cd_4_Te_5_O_14_

**DOI:** 10.1107/S2056989024010387

**Published:** 2024-11-05

**Authors:** Felix Eder, Matthias Weil

**Affiliations:** a TU Wien, Institute for Chemical Technologies and Analytics, Division of Structural Chemistry, Getreidemarkt 9/E164-05-1, 1060 Vienna, Austria; University of Kentucky, USA

**Keywords:** crystal structure, cadmium, tellurite, nitrate, layer structure, electron lone pair

## Abstract

Monoclinic single crystals of Cd_5_(TeO_3_)_4_(NO_3_)_2_ (space group *P*2_1_/*c*), penta­cadmium tetra­kis­[oxidotellurate(IV)] dinitrate, and of Cd_4_Te_5_O_14_ (space group *C*2/*c*), tetra­cadmium penta­oxidotellurate(IV), were obtained under the same hydro­thermal conditions. Whereas the crystal structure of Cd_5_(TeO_3_)_4_(NO_3_)_2_ is distinctively layered, that of Cd_4_Te_5_O_14_ exhibits a tri-periodic framework. In Cd_5_(TeO_3_)_4_(NO_3_)_2_, the three Cd^II^ atoms have coordination numbers (CN) of 7, 6 and 6. The two types of [CdO_6_] and the [CdO_7_] polyhedra [bond lengths range from 2.179 (3) to 2.658 (2) Å] share corners and edges, resulting in layers extending parallel to (100). Both Te^IV^ atoms are coordinated by three oxygen atoms in a trigonal–pyramidal shape. The oxygen atoms of the isolated [TeO_3_] groups [bond lengths range from 1.847 (3) to 1.886 (3) Å] all are part of the cadmium–oxygen layer. The electron lone pairs ψ of the Te^IV^ atoms are directed away from the layer on both sides. The available inter­layer space is co-occupied by the nitrate group, which is directly connected with two of its O atoms to the layer whereas the third O atom is solely bonded to the N atom and points towards the adjacent layer. In Cd_4_Te_5_O_14_, all three unique Cd^II^ atoms are coordinated by six oxygen atoms, considering Cd—O distances from 2.235 (2) to 2.539 (2) Å. By edge- and corner-sharing, the distorted [CdO_6_] polyhedra form an open framework that is partially filled with three different stereochemically active Te^IV^ atoms. All of them exhibit a CN of 4, with Te—O bonds in a range from 1.859 (2) to 2.476 (2) Å. The corresponding [TeO_4_] units are linked to each other by corner- and edge-sharing, forming infinite helical ^1^_∞_[Te_10_O_28_] chains extending parallel to [203]. The connectivity in the chains can be described as (⋯–⋄–⋄=⋄–⋄–⋄–⋄–⋄=⋄–⋄–⋄–⋯)_*n*_ where ‘⋄’ denotes a [TeO_4_] unit, ‘–’ a linkage *via* corners and ‘=’ a linkage *via* edges. Such a structural motif is unprecedented in the crystal chemistry of oxidotellurate(IV) compounds.

## Abstract 2

The crystal structure of Cd_5_(TeO_3_)_4_(NO_3_)_2_ exhibits a distinct layered arrangement, whereas Cd_4_Te_5_O_14_ crystallizes with a framework structure.

## 1. Chemical context

Oxidotellurates show a vast structural diversity, in particular with tellurium in the +IV oxidation state, which has been summarized and categorized recently (Christy *et al.*, 2016 ▸). This variety can be attributed to the different coordination numbers (CNs) of the Te^IV^ atom to the oxygen ligands (ranging from 3 to 5 for the first coordination sphere) and, particularly, to the 5*s*^2^ electron lone pair (ψ) localized at the Te^IV^ atom. The large space consumption of ψ often leads to rather low-symmetric and one-sided anionic coordination polyhedra in oxidotellurates(IV), and in consequence to the formation of modular structural motifs like clusters, chains, layers, or to open-frameworks penetrated by channels (Stöger & Weil, 2013 ▸). These features can be enhanced by introducing other oxido-anion groups as spacers into the oxidotellurate(IV) framework. This concept has already proven successful for several types of oxido-anion groups, for example in the form of tetra­hedral groups like in sulfates or selenates (Weil & Shirkhanlou, 2017 ▸), phosphates (Eder & Weil, 2020*a* ▸), and arsenates (Missen *et al.*, 2020 ▸), or in the form of trigonal–planar groups like in carbonates (Eder *et al.*, 2022 ▸) and nitrates (Lee *et al.*, 2021 ▸; Stöger & Weil, 2013 ▸).

For the present study, we have focused on divalent metal oxidotellurates(IV) modified by nitrate anions. For this purpose, we have used our experience with the system Ca–Te–O, for which corresponding phases such as Ca_5_Te_4_O_12_(NO_3_)_2_(H_2_O)_2_ and Ca_6_Te_5_O_15_(NO_3_)_2_ exist (Stöger & Weil, 2013 ▸). Since the ionic radii of Ca^II^ and Cd^II^ differ only slightly (Shannon, 1976 ▸), the Cd–Te–O system appears promising in this regard. In fact, we were able to hydro­thermally grow single crystals of corresponding cadmium oxidotellurate(IV) nitrate phases with the composition Cd_5_(TeO_3_)_4_(NO_3_)_2_ and Cd_4_Te_4_O_11_(NO_3_)_2_ (Eder, 2023 ▸). Under the same hydro­thermal conditions, single crystals of the nitrate-free compound Cd_4_Te_5_O_14_ were also obtained as a minor by-product, next to other impurity phases.

In the present communication we report on preparation conditions and crystal structures of Cd_5_(TeO_3_)_4_(NO_3_)_2_ and Cd_4_Te_5_O_14_. As a result of systematic twinning and the resulting problems in the processing of the diffraction intensities, the crystal structure refinement of Cd_4_Te_4_O_11_(NO_3_)_2_ must be regarded as unsatisfactory. The preliminary structure model is deposited in form of a Crystallographic Information File (CIF; Hall *et al.*, 2006 ▸) and is available from the electronic supporting information (ESI) of this article.

## 2. Structural commentary

**Cd_5_(TeO_3_)_4_(NO_3_)_2_**

The asymmetric unit comprises two Te, three Cd, one N and nine O atoms. With the exception of Cd3 (site symmetry 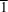; multiplicity 2, Wyckoff letter *a*), all atoms are located at sites corresponding to general positions (4 *e*) of space group *P*2_1_/*c*.

**Figure 1:**
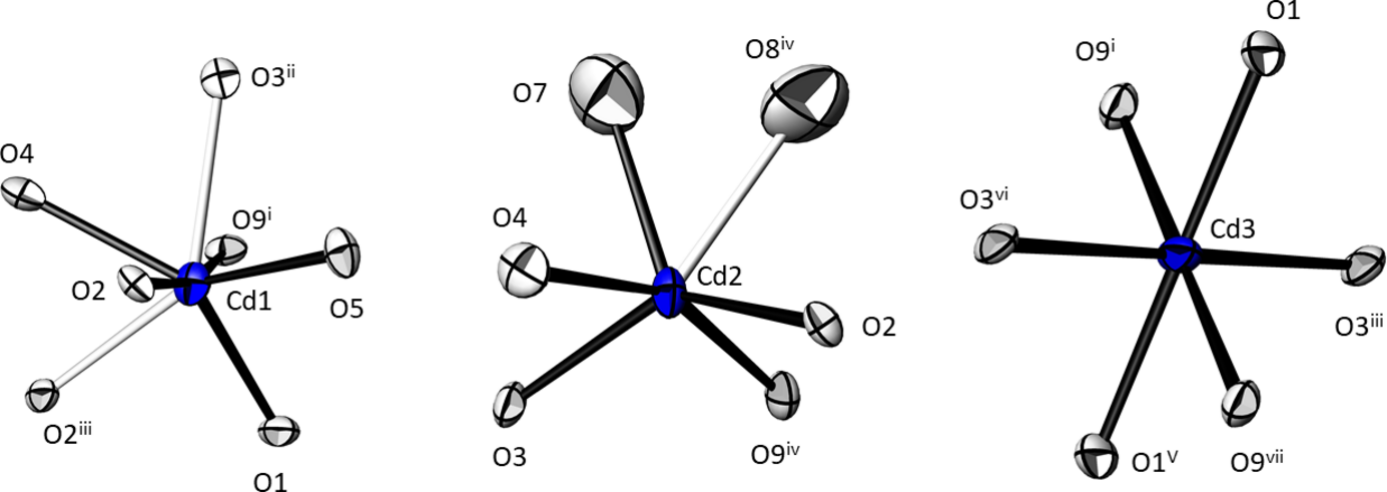
The three different [CdO_*x*_] polyhedra in the crystal structure of Cd_5_(TeO_3_)_4_(NO_3_)_2_. Bonds shorter than 2.50 Å are black, and white for longer Cd—O contacts. Displacement ellipsoids are drawn at the 74% probability level; symmetry codes refer to Table 1 ▸.

**Figure 2:**
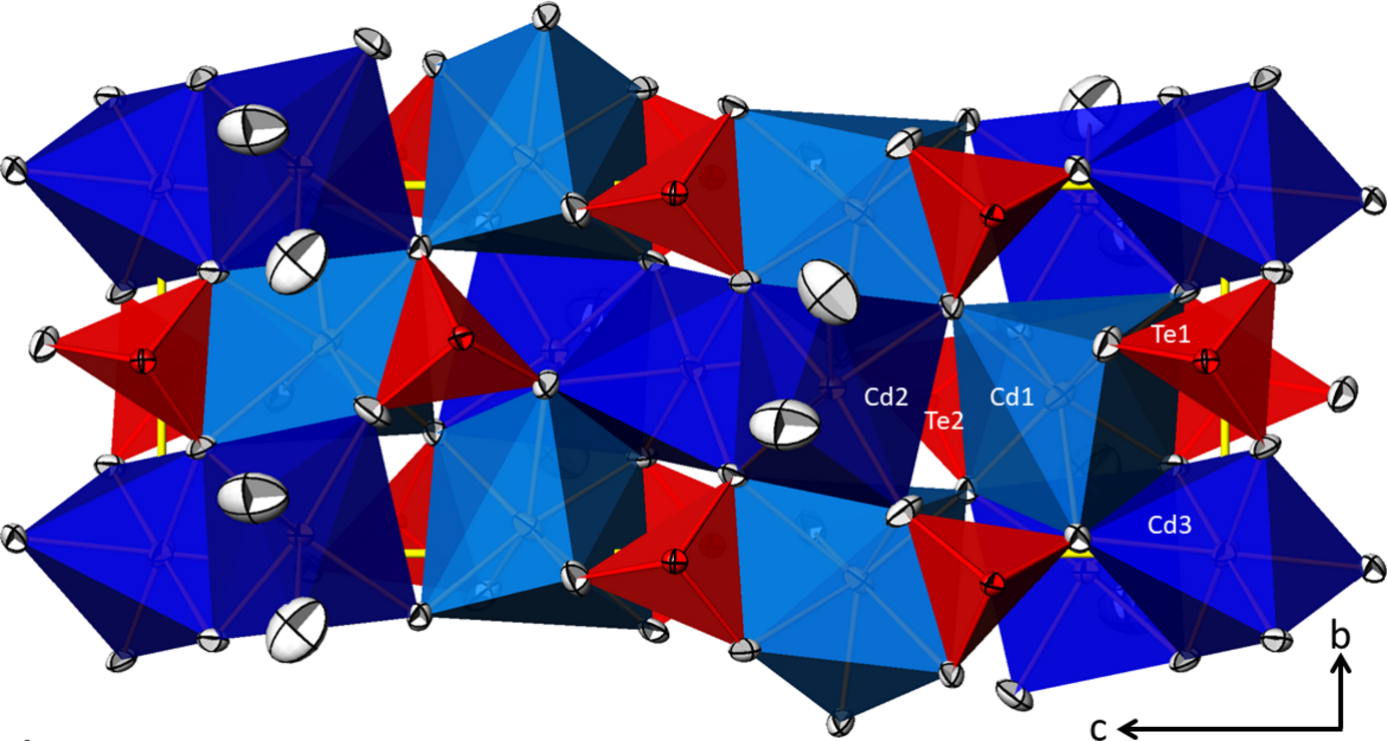
View on top of one [Cd–Te–O] layer in the crystal structure of Cd_5_(TeO_3_)_4_(NO_3_)_2_. Displacement ellipsoids are drawn at the 74% probability level; nitrate groups and electron lone pairs are not shown for clarity.

The Cd^II^ atoms exhibit a CN of [5 + 2] for Cd1, [5 + 1] for Cd2, and 6 for Cd3 when the inner coordination sphere is comprised of oxygen atoms at distances between 2.179 (3) and 2.389 (5) Å, and the outer coordination sphere at distances between 2.524 (3) and 2.658 (2) Å (Table 1 ▸, Fig. 1 ▸). Including these remote oxygen atoms for the bond valence sum (BVS) calculations (Brown, 2002 ▸), the values for the Cd^II^ atoms amount to 1.92 (Cd1), 2.02 (Cd2) and 2.09 (Cd3) valence units (v. u.) using the parameters of Brese & O’Keeffe (1991 ▸). The mean Cd—O distances are 2.403 Å for Cd1, 2.328 Å for Cd2, and 2.295 Å for Cd3, in good agreement with literature values [2.302 (69) Å for CN = 6 from 135 coordination polyhedra, 2.377 (134) Å for CN = 7 from 6 polyhedra; Gagné & Hawthorne, 2020 ▸]. The two [CdO_6_] and the [CdO_7_] polyhedra are connected by corner- and edge-sharing and thereby form layers extending parallel to (100) (Fig. 2 ▸).

Both Te sites are coordinated by three oxygen atoms in a trigonal-pyramidal shape. If the non-bonding 5*s*^2^ electron lone pair ψ of the Te^IV^ atoms is also taken into account, [ψTeO_3_] polyhedra with the shapes of flattened tetra­hedra are formed. The positions of ψ were calculated with the *LPLoc* program (Hamani *et al.*, 2020 ▸) resulting in the following fractional coordinates: *x* = 0.3787, *y* = 0.4979, *z* = 0.0221 for ψ1 at the Te1 atom (distance Te1—ψ1 = 1.195 Å**)**, and *x* = 0.6617, *y* = 0.3689, *z* = 0.2780 for ψ2 at the Te2 atom (distance Te2—ψ2 = 1.202 Å**)**. The [TeO_3_] units are isolated from each other with a connectivity of Q^3000^ according to the notation of Christy *et al.* (2016 ▸). The BVS values of the Te^IV^ atoms using the parameters of Mills & Christy (2013 ▸) closely correspond to the expectation of 4 v. u., with values of 3.91 (Te1) and 4.06 (Te2) v.u.

**Figure 3:**
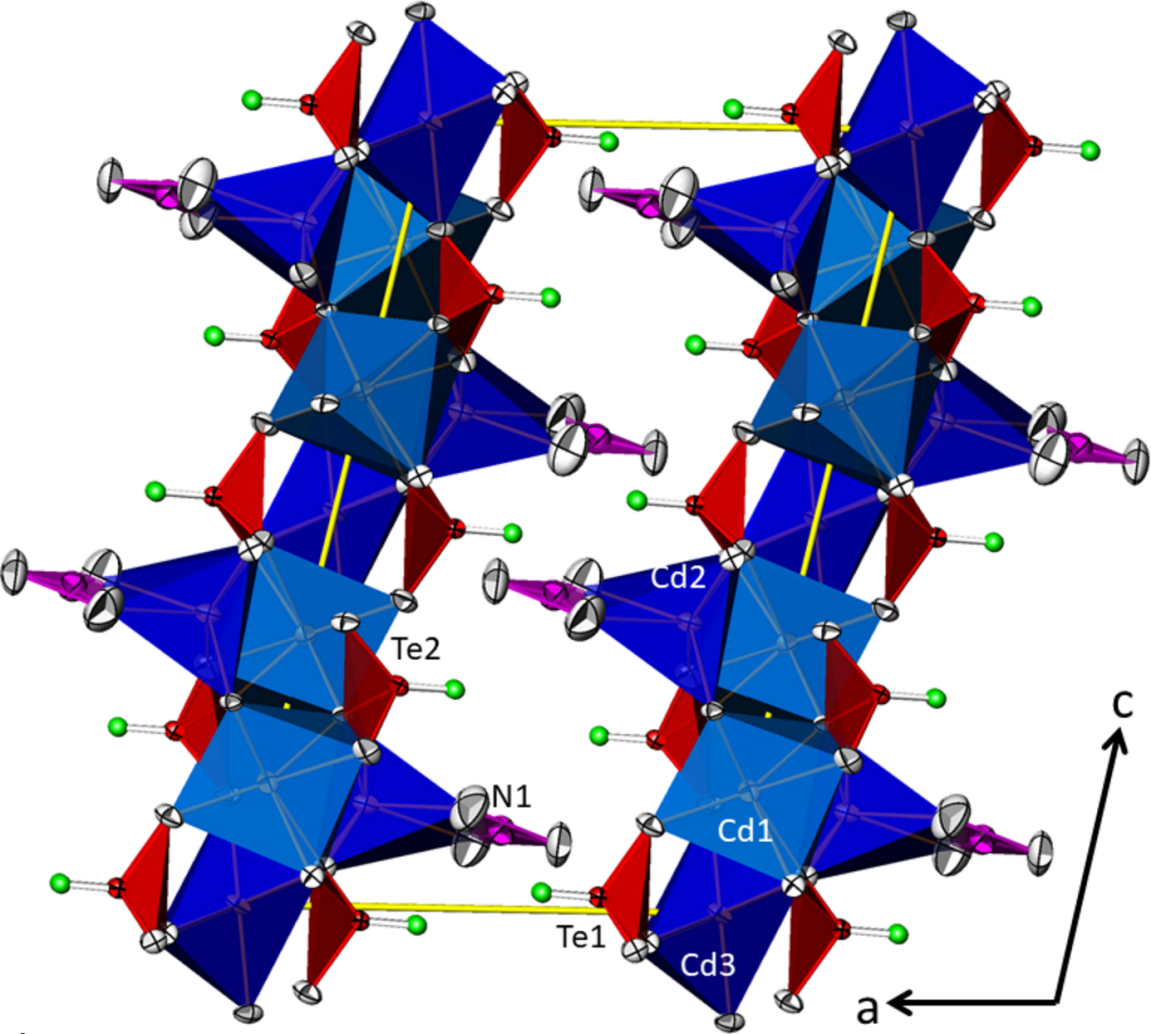
The layered arrangement of Cd_5_(TeO_3_)_4_(NO_3_)_2_ as seen in a projection along [0
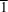
0]. Displacement ellipsoids are drawn at the 74% probability level, and electron lone pairs are shown as green spheres with arbitrary size.

The [TeO_3_] groups are part of the cadmium–oxygen layers mentioned above (Fig. 2 ▸). The electron lone pairs ψ of both Te^IV^ atoms are directed away from the layer on both sides (Fig. 3 ▸). Next to the free electron pairs ψ, the space available between adjacent layers is partly co-occupied by the nitrate group (N1, O6, O7 and O8). The (NO_3_)^−^ anion is bound to the layer by sharing an edge with the [Cd2O_6_] polyhedron with one shorter contact [2.389 (5) Å to O7] and one longer contact [2.587 (5) Å to O8] to the Cd2 atom. The third oxygen atom of the nitrate anion, O6, is not in the coordination sphere of any Cd^II^ atom and has a slightly shorter N—O bond length of 1.234 (5) Å compared to the other two [1.242 (6) and 1.245 (6) Å]. The average N—O bond length amounts to 1.240 Å and closely matches the literature value of 1.247 (29) Å calculated for 468 N—O bonds in the nitrate anion (Gagné & Hawthorne, 2020 ▸). The O—N—O bond angles range from 117.6 (5)° (for the O atoms sharing edges with the [Cd2O_6_] unit) to 121.6 (5)°, indicating a slight angular distortion. The (NO_3_)^−^ group deviates from planarity, as observed for many nitrates with deviations of up to 0.02 Å (Jarosch & Zemann, 1983 ▸). In Cd_5_(TeO_3_)_4_(NO_3_)_2_, the root-mean-square deviation of the four atoms of the (NO_3_)^−^ group is 0.0082 Å, with a deviation for N1 of −0.014 (4) Å from the plane defined by O6, O7(−*x* + 1, *y* − 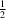, −*z* + 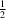) and O8. The weak binding of the nitrate group to the layers is reflected in its significantly larger displacement parameters compared to the other atoms of the network (Fig. 3 ▸).

While there are no phases isotypic with Cd_5_(TeO_3_)_4_(NO_3_)_2_ known so far, the calcium compound Ca_5_(TeO_3_)_4_(NO_3_)_2_(H_2_O)_2_ (space group *Cc*, *Z* = 4; Stöger & Weil, 2013 ▸) shows some similarities with the cadmium compound. Ca_5_(TeO_3_)_4_(NO_3_)_2_(H_2_O)_2_ consists of (100) [Ca–Te–O] layers that are built in the same way as the [Cd–Te–O] layers in Cd_5_(TeO_3_)_4_(NO_3_)_2_. This is reflected in similar lattice parameters *b* and *c* for both phases. The slightly longer axes, *b* = 5.7289 (7) Å and *c* = 17.007 (2) Å for Ca_5_(TeO_3_)_4_(NO_3_)_2_(H_2_O)_2_ compared to *b* = 5.6173 (2) Å and *c* = 16.6136 (7) Å for Cd_5_(TeO_3_)_4_(NO_3_)_2_, are primarily caused by the larger ionic radii (Shannon, 1976 ▸) of Ca^II^ (1.00 Å for CN 6, 1.06 Å for CN 7) compared to Cd^II^ (0.93 Å for CN 6, 1.03 Å for CN 7). The main differences between the two structures originate from the space between the layers. In the crystal structure of Ca_5_(TeO_3_)_4_(NO_3_)_2_(H_2_O)_2_, the two water mol­ecules are tightly bound to one of the Ca^II^ atoms with Ca—O bond lengths of 2.390 (9) and 2.39 (2) Å. The nitrate groups, however, are not connected that well to the framework like in the crystal structure of the cadmium compound. One nitrate group shares one corner with the layer [Ca—O distance = 2.462 (11) Å], while the other (NO_3_)^−^ anion is completely isolated from the layers. The more loosely bound (NO_3_)^−^ group can be seen as the main reason why Ca_5_(TeO_3_)_4_(NO_3_)_2_(H_2_O)_2_ exhibits diffuse scattering caused by stacking disorder, which can be described by OD theory (Dornberger-Schiff & Grell-Niemann, 1961 ▸; Stöger & Weil, 2013 ▸). On the contrary, no signs of diffuse scattering were discernible in the diffraction pattern of Cd_5_(TeO_3_)_4_(NO_3_)_2_.

**Cd_4_Te_5_O_14_**

Of the thirteen atoms in the asymmetric unit, three (Cd2, Cd3, Te3) are located at positions with site symmetry 2 (4 *e*), while the other ten (two Te, one Cd and seven O) all belong to general 8 *f* positions of space group *C*2/*c*.

**Figure 4:**
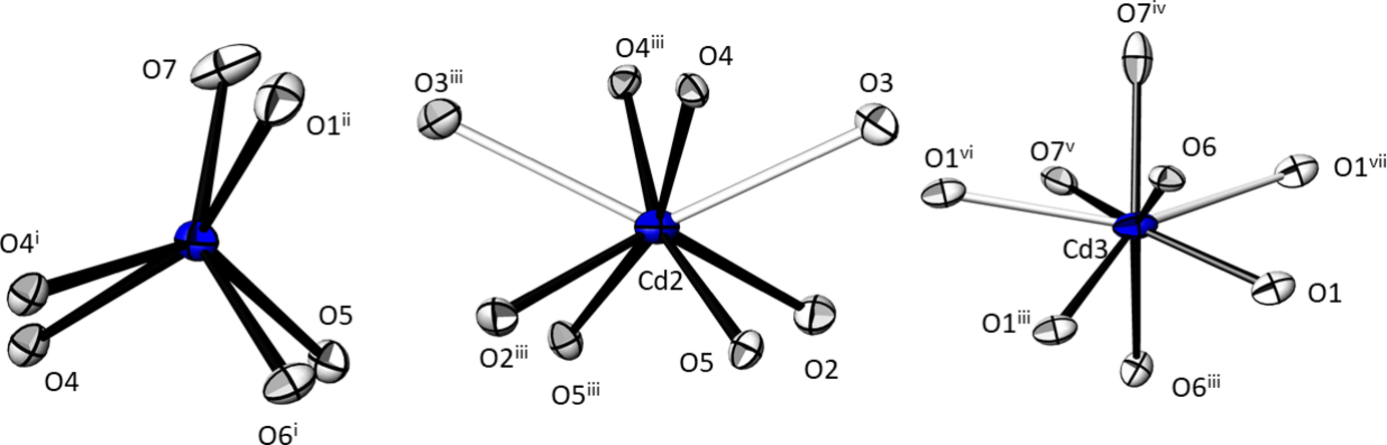
The three different [CdO_*x*_] polyhedra in the crystal structure of Cd_4_Te_5_O_14_. Bonds shorter than 2.50 Å are black, and white for longer Cd—O contacts. Displacement ellipsoids are drawn at the 74% probability level; symmetry codes refer to Table 2 ▸.

The three Cd^II^ atoms are coordinated by six O atoms with distances between 2.235 (2) and 2.539 (2) Å (Table 2 ▸). The [Cd1O_6_] polyhedron has a distorted trigonal-prismatic shape, while the [Cd2O_6_] and [Cd3O_6_] units have rather irregular shapes. In both cases, this might be caused by the presence of two additional oxygen contacts at distances of 2.809 (3) Å for Cd2 and of 2.860 (3) Å for Cd3, respectively. Hence, the coordination numbers of the Cd^II^ atoms are best described as 6 for Cd1 and [6 + 2] for Cd2 and Cd3 (Fig. 4 ▸) The meaningfulness to include the remote oxygen atoms is underlined by the BVS of the Cd^II^ atoms using the parameters of Brese & O’Keeffe (1991 ▸). Based on sixfold coordination, these values amount to 2.00 (Cd1), 1.79 (Cd2) and 1.71 (Cd3) v. u. The latter two values increase to 1.97 (Cd2) and 1.86 (Cd3) v. u. under consideration of the two additional oxygen atoms. Likewise, the mean Cd—O distances, 2.323 Å for Cd1 (CN = 6), 2.456 Å for Cd2 (CN = 8) and 2.497 Å for Cd3 (CN = 8), comply with literature values (for Cd—O with CN = 6 (*vide supra*); 2.432 (118) Å for CN = 8 from 18 polyhedra; Gagné & Hawthorne, 2020 ▸).

The Te^IV^ atoms are all coordinated by four oxygen atoms in bis­phenoidal shapes. While for Te3 the four oxygen contacts have comparable distances, for Te1 and Te2 the coordination is better described as [3 + 1] (Table 2 ▸). It should be noted that the fourth oxygen contact of Te1 has a distance of 2.476 (2) Å to O2^iii^ and thus is slightly above the bond-length threshold of 2.40–2.45 Å. The latter was suggested by Christy *et al.* (2016 ▸) to distinguish between ‘structural unit’ and ‘inter­stitial complex’ (Hawthorne, 2014 ▸). However, the BVS of Te1 is perfectly defined with the parameters of Brese & O’Keeffe (1991 ▸), resulting in a value of 4.00 v.u. compared to 3.74 v.u. without the fourth O atom. Hence, Te1 was considered as fourfold-coordinated as well. The BVS of the three Te^IV^ atoms amount to 4.01 (Te1), 3.93 (Te2) and 4.00 (Te3) v.u. when applying the revised parameters (Mills & Christy, 2013 ▸). The lone-pair electrons of the Te^IV^ atoms are stereochemically active and point away from the backbone of the bis­phenoids in each case. The fractional coordinates of ψ were computed with *LPLoc* (Hamani *et al.*, 2020 ▸) and amount to *x* = 0.14556, *y* = 0.07878, *z* = −0.02783 for ψ1 at the Te1 atom (distance Te1—ψ1 = 0.968 Å), *x* = 0.23325, *y* = 0.35725, *z* = 0.15340 for ψ2 at the Te2 atom (distance Te2—ψ2 = 0.932 Å), and *x* = 0, *y* = 0.85614, *z* = 0.25 for ψ3 at the Te3 atom (distance Te3—ψ3 = 1.356 Å).

**Figure 5:**
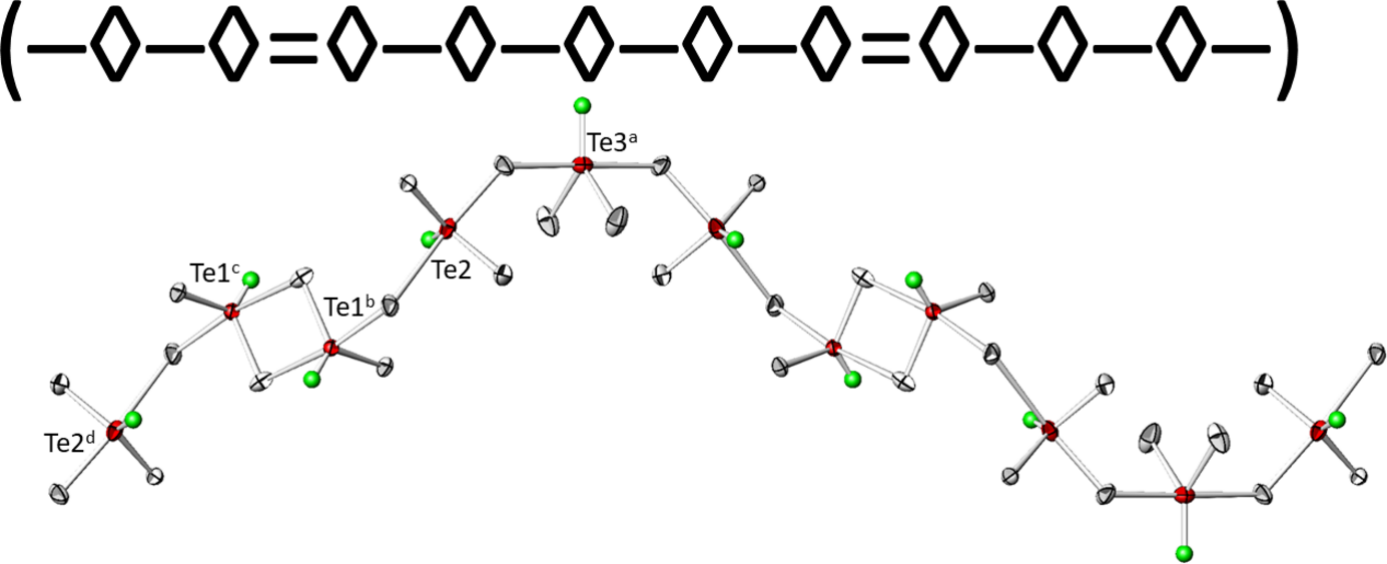
The ^1^_∞_[Te_10_O_28_] chain in the crystal structure of Cd_4_Te_5_O_14_. In the graphical representation, ‘⋄’ denotes a [TeO_4_] unit, ‘–’ a linkage *via* corners and ‘=’ a linkage *via* edges. Displacement ellipsoids are drawn at the 74% probability level, and electron lone pairs are shown as green spheres with arbitrary size. [Symmetry codes: (a) *x*, 1 − *y*, −*z*; (b) 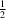 − *x*, 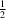 + *y*, 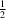 − *z*; (c) 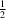 + *x*, 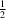 − *y*, 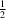 + *z*; (d) 1 − *x*, 1 − *y*, 1 − *z*.]

**Figure 6:**
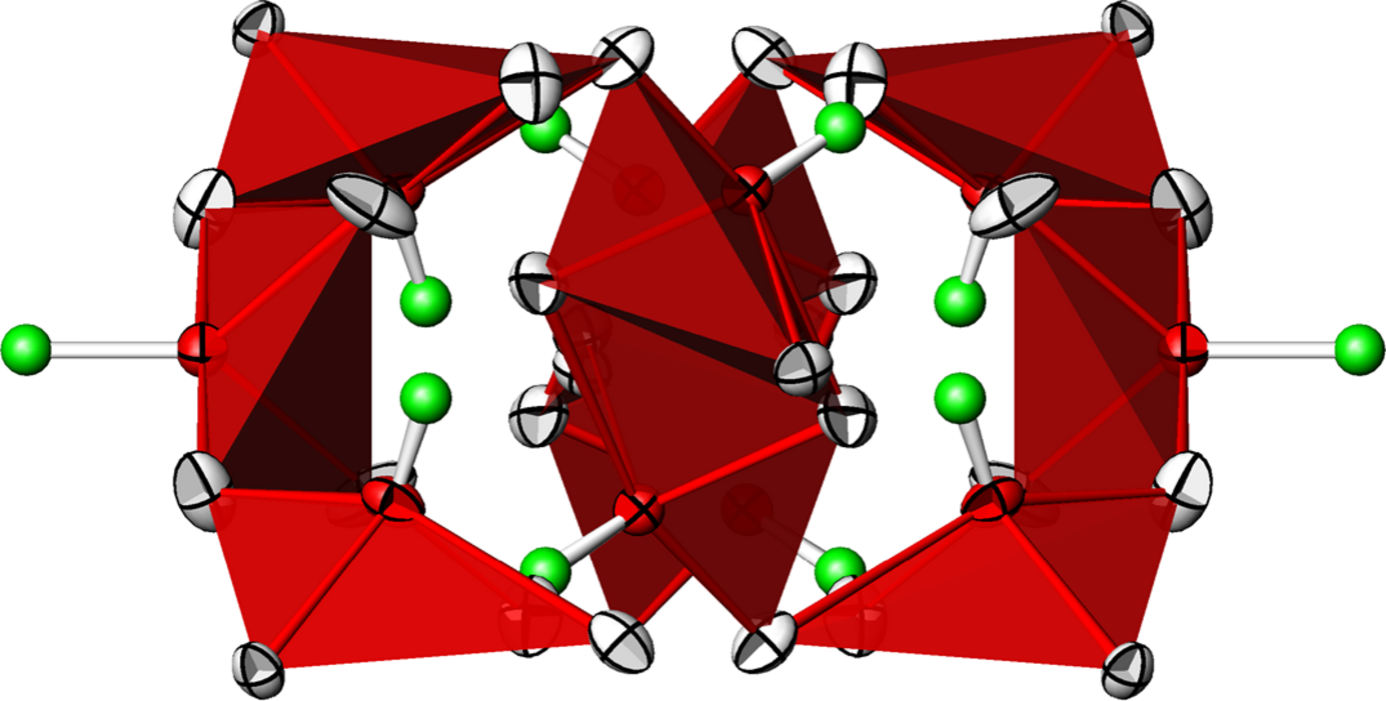
Projection along [203] of the ^1^_∞_[Te_10_O_28_] chain in polyhedral representation, with the [TeO_4_] units shown in red.

**Figure 7:**
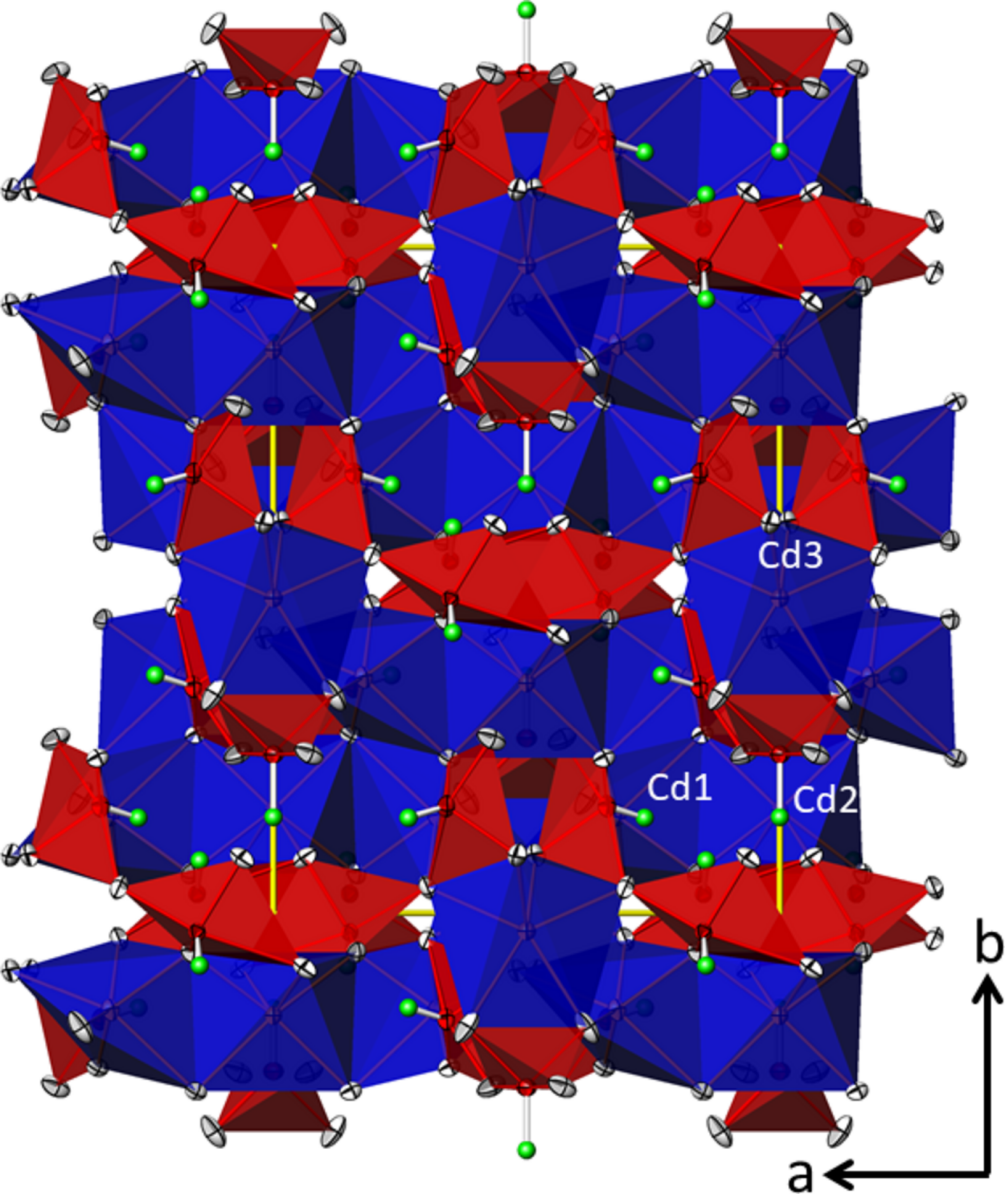
The framework structure of Cd_4_Te_5_O_14_ in a view along [00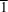]. For clarity, the two longer Cd⋯O contacts of Cd2 and Cd3 were not used to define the polyhedra, which are displayed with CN = 6. Displacement ellipsoids are drawn at the 74% probability level, and electron lone pairs are shown as green spheres with an arbitrary size.

The three [TeO_4_] units are connected to each other forming _∞_^1^[Te_10_O_24/2_O_16/1_] chains propagating parallel to [203] (Fig. 5 ▸), corresponding to a translation of 2**a** + 3**c**. The translational period of the chain is ten Te^IV^ atoms long and consequently the oxidotellurate(IV) chains are categorized as *zehner*-chains (Liebau, 1985 ▸). Using the nomenclature of Christy *et al.* (2016 ▸), the connectivities of the three Te^IV^ atoms are denoted as Q^1301^ (Te1) and Q^2200^ (Te2 and Te3), with a graphical representation of (⋯–⋄–⋄=⋄–⋄–⋄–⋄–⋄=⋄–⋄–⋄–⋯), where ‘⋄’ denotes a [TeO_4_] unit, ‘–’ a linkage *via* corners and ‘=’ a linkage *via* edges. The chains form loops leading to the shape of an ‘∞’ when viewed along the propagating direction (Fig. 6 ▸). This structural element has not been described yet for oxidotellurates (Christy *et al.*, 2016 ▸). The ^1^_∞_[Te_10_O_28_] chains share their oxygen atoms with the Cd^II^ atoms to form a rather dense framework structure (Fig. 7 ▸).

Unlike *β*-CdTe_2_O_5_ (Eder & Weil, 2020*b* ▸), which is isotypic with the corresponding Ca-compound (Weil & Stöger, 2008 ▸), the crystal structure of Cd_4_Te_5_O_14_ has no structural relationship with the two polymorphs of the corresponding Ca-compound, Ca_4_Te_5_O_14_. In *α*-Ca_4_Te_5_O_14_ (Weil, 2004 ▸), the Te^IV^ atoms form branched [Te_8_O_22_] *achter*-single chains (⋯–(⋄–Δ)–⋄–⋄–(⋄–Δ)–⋄–⋄–⋯) as well as isolated [TeO_3_] (Δ) groups. The high-pressure *β*-polymorph (Weil *et al.*, 2016 ▸) consists of isolated [Te_3_O_8_] (Δ–⋄–Δ) and [TeO_3_] (Δ) units. If a Te—O contact of 2.479 (2) Å is considered as well, two [Te_3_O_8_] units are connected to form a [Te_6_O_16_] group (Δ–⋄–⋄–⋄–⋄–Δ).

## 3. Synthesis and crystallization

Hydro­thermal experiments were carried out at a temperature of 483 K with a reaction time of one week. The reaction containers were small Teflon vessels with an inner volume of *ca*. 3–4 ml. The educts, generally a total of 0.5–1 g, were weighed and mixed dry manually with a spatula in the vessels. Afterwards, water was added until the reaction container was filled to *ca*. 2/3 of its volume, and the mixture was manually stirred again. The Teflon containers were then placed into steel autoclaves and transferred to a preheated drying oven. Cooling was achieved within circa 3 h by taking the autoclaves out of the oven.

Initially, Cd_5_(TeO_3_)_4_(NO_3_)_2_ was obtained in a hydro­thermal reaction starting from Cd(NO_3_)_2_(H_2_O)_4_, TeO_2_ and KOH (molar ratios 2:1:2). Other hydro­thermal experiments aimed at the repeated synthesis of Cd_5_(TeO_3_)_4_(NO_3_)_2_, but started from different ratios of Cd(NO_3_)_2_(H_2_O)_4_ and either K_2_TeO_3_ or KOH and TeO_2_, and were performed under the described hydro­thermal conditions or without any additional water. Cd_5_(TeO_3_)_4_(NO_3_)_2_ was obtained in seven of the twelve batches. In three of these batches, a second cadmium oxidotellurate(IV) nitrate phase with presumed composition Cd_4_Te_4_O_11_(NO_3_)_2_ and a likewise layered structural arrangement was obtained. Crystal structure refinement of this phase was hampered by systematic twinning and overlapping reflections. The preliminary model given in the ESI is based on overlapping intensity data of a multi-domain crystal and a primitive triclinic unit cell (*Z* = 2, *a* ≃ 9.42, *b* ≃ 9.43, *c* ≃ 9.61 Å, *α* ≃ 92, *β* ≃ 108, *γ* ≃ 109°).

Yet another new phase, Cd_4_Te_5_O_14_, was isolated in form of single crystals from one of these batches, using a molar ratio of Cd(NO_3_)_2_(H_2_O)_4_:K_2_TeO_3_ = 2:1. This phase has formed in only small amounts, because powder X-ray diffraction (PXRD) measurements of the bulk material revealed a negligible fraction of this phase relative to the other products. In all batches, phase-mixtures were present. Next to the three new compounds, Cd_2_Te_3_O_9_ (Weil, 2004 ▸), *α*-TeO_2_ (Thomas, 1988 ▸), *β*-Cd_3_TeO_6_ (Weil & Veyer, 2018 ▸) and CdTeO_3_ (Krämer & Brandt, 1985 ▸) could be detected in the washed products. However, some reflections could not be assigned to any of the known phases stored in the Powder Diffraction File (PDF-4; Gates-Rector & Blanton, 2019 ▸). It should also be mentioned that reflections assignable to Cd_5_(TeO_3_)_4_(NO_3_)_2_ exhibited a preferred orientation of the (*n*00) planes, which is not surprising since the crystal structure shows an assembly of (100) layers.

Single crystals of Cd_5_(TeO_3_)_4_(NO_3_)_2_ are colorless and have the form of elongated plates whereas single crystals of Cd_4_Te_5_O_14_ are colourless and bar-shaped. Both types of crystals were manually isolated from the bulk products and subjected to single crystal X-ray studies.

## 4. Refinement

Crystal data, data collection and structure refinement details are summarized in Table 3 ▸. Structure data of both title compounds were standardized with *STRUCTURE-TIDY* (Gelato & Parthé, 1987 ▸). For refinement of Cd_5_(TeO_3_)_4_(NO_3_)_2_, one reflection (100) was obstructed by the beamstop and was omitted from the data.

## Supplementary Material

Crystal structure: contains datablock(s) Cd4Te5O14, Cd5TeO34NO32, general. DOI: 10.1107/S2056989024010387/pk2712sup1.cif

Structure factors: contains datablock(s) Cd4Te5O14. DOI: 10.1107/S2056989024010387/pk2712Cd4Te5O14sup2.hkl

Structure factors: contains datablock(s) Cd5TeO34NO32. DOI: 10.1107/S2056989024010387/pk2712Cd5TeO34NO32sup3.hkl

CCDC references: 2393449, 2393448

Additional supporting information:  crystallographic information; 3D view; checkCIF report

## Figures and Tables

**Table 1:** Selected bond lengths (Å) for Cd_5_(TeO_3_)_4_(NO_3_)_2_

Cd1—O5	2.281 (3)	Cd3—O1^v^	2.269 (3)
Cd1—O2	2.292 (3)	Cd3—O3^vi^	2.289 (3)
Cd1—O1	2.326 (3)	Cd3—O3^iii^	2.289 (3)
Cd1—O9^i^	2.361 (3)	Cd3—O9^vii^	2.326 (3)
Cd1—O4^ii^	2.380 (3)	Cd3—O9^i^	2.326 (3)
Cd1—O2^iii^	2.524 (3)	Te1—O5	1.847 (3)
Cd1—O3^ii^	2.658 (3)	Te1—O3^vi^	1.875 (3)
Cd2—O4	2.179 (3)	Te1—O9^viii^	1.886 (3)
Cd2—O9^iv^	2.256 (3)	Te2—O1^iv^	1.858 (3)
Cd2—O2	2.261 (3)	Te2—O4^iv^	1.869 (3)
Cd2—O3	2.296 (3)	Te2—O2^ix^	1.886 (3)
Cd2—O7	2.389 (5)	N1—O6	1.234 (5)
Cd2—O8^iv^	2.587 (5)	N1—O7^ix^	1.242 (6)
Cd3—O1	2.269 (3)	N1—O8	1.245 (6)

Symmetry codes: (i) 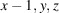; (ii) 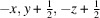; (iii) 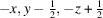; (iv) 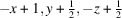; (v) 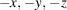; (vi) 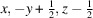; (vii) 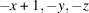; (viii) 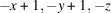; (ix) 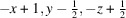.

**Table 2:** Selected bond lengths (Å) for Cd_4_Te_5_O_14_

Cd1—O7	2.235 (2)	Cd3—O6	2.330 (2)
Cd1—O4^i^	2.237 (2)	Cd3—O7^iv^	2.478 (3)
Cd1—O5	2.262 (2)	Cd3—O7^v^	2.478 (3)
Cd1—O1^ii^	2.314 (2)	Cd3—O1^vi^	2.860 (2)
Cd1—O6^i^	2.353 (2)	Cd3—O1^vii^	2.860 (2)
Cd1—O4	2.539 (2)	Te1—O2^viii^	1.873 (2)
Cd2—O4^iii^	2.327 (2)	Te1—O5	1.882 (2)
Cd2—O4	2.327 (2)	Te1—O6^ix^	1.936 (2)
Cd2—O5^iii^	2.339 (2)	Te1—O2^iii^	2.476 (2)
Cd2—O5	2.339 (2)	Te2—O1	1.859 (2)
Cd2—O2^iii^	2.395 (2)	Te2—O4	1.890 (2)
Cd2—O2	2.395 (2)	Te2—O3	1.928 (2)
Cd2—O3	2.809 (2)	Te2—O6	2.441 (2)
Cd2—O3^iii^	2.809 (2)	Te3—O7^v^	1.876 (2)
Cd3—O1^iii^	2.318 (2)	Te3—O7^iv^	1.876 (2)
Cd3—O1	2.318 (2)	Te3—O3^vi^	2.088 (2)
Cd3—O6^iii^	2.330 (2)	Te3—O3^vii^	2.088 (2)

Symmetry codes: (i) 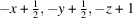; (ii) 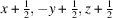; (iii) 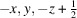; (iv) 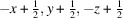; (v) 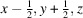; (vi) 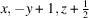; (vii) 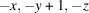; (viii) 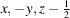; (ix) 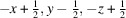.

**Table 3:** Experimental details

	Cd_5_(TeO_3_)_4_(NO_3_)_2_	Cd_4_Te_5_O_14_
Crystal data		
*M*_r_	1388.42	1311.60
Crystal system, space group	Monoclinic, *P*2_1_/*c*	Monoclinic, *C*2/*c*
Temperature (K)	300	296
*a*, *b*, *c* (Å)	9.9442 (4), 5.6173 (2), 16.6136 (7)	11.9074 (3), 14.3289 (3), 8.7169 (2)
β (°)	102.737 (1)	113.629 (1)
*V* (Å^3^)	905.19 (6)	1362.58 (6)
*Z*	2	4
Radiation type	Mo *K*α	Mo *K*α
μ (mm^−1^)	12.19	16.73
Crystal size (mm)	0.08 × 0.03 × 0.02	0.09 × 0.07 × 0.06

Data collection		
Diffractometer	Bruker APEXII CCD	Bruker APEXII CCD
Absorption correction	Multi-scan (*SADABS*; Krause *et al.*, 2015 ▸)	Multi-scan (*SADABS*; Krause *et al.*, 2015 ▸)
*T*_min_, *T*_max_	0.517, 0.747	0.595, 0.747
No. of measured, independent and observed [*I* > 2σ(*I*)] reflections	17368, 4388, 3381	18799, 3373, 3084
*R*_int_	0.050	0.027
(sin θ/λ)_max_ (Å^−1^)	0.834	0.839

Refinement		
*R*[*F*^2^ > 2σ(*F*^2^)], *wR*(*F*^2^), *S*	0.030, 0.061, 0.99	0.022, 0.040, 1.21
No. of reflections	4388	3373
No. of parameters	133	106
Δρ_max_, Δρ_min_ (e Å^−3^)	1.56, −2.37	1.10, −1.53

Computer programs: *APEX4* (Bruker, 2021 ▸), *APEX3* and *SAINT* (Bruker, 2016 ▸), *SHELXT* (Sheldrick, 2015*a* ▸), *SHELXL* (Sheldrick, 2015*b* ▸), *ATOMS* (Dowty, 2006 ▸) and *publCIF* (Westrip, 2010 ▸).

## supplementary crystallographic information

### Pentacadmium tetrakis[oxidotellurate(IV)] dinitrate (Cd4Te5O14) Crystal data

**Table:**

Cd_4_Te_5_O_14_	*F*(000) = 2256
*M**_r_* = 1311.60	*D*_x_ = 6.394 Mg m^−^^3^
Monoclinic, *C*2/*c*	Mo *K*α radiation, λ = 0.71073 Å
*a* = 11.9074 (3) Å	Cell parameters from 8153 reflections
*b* = 14.3289 (3) Å	θ = 2.4–36.6°
*c* = 8.7169 (2) Å	µ = 16.73 mm^−^^1^
β = 113.629 (1)°	*T* = 296 K
*V* = 1362.58 (6) Å^3^	Fragment, colourless
*Z* = 4	0.09 × 0.07 × 0.06 mm

### Pentacadmium tetrakis[oxidotellurate(IV)] dinitrate (Cd4Te5O14) Data collection

**Table:**

Bruker APEXII CCD diffractometer	3084 reflections with *I* > 2σ(*I*)
ω– and φ–scan	*R*_int_ = 0.027
Absorption correction: multi-scan (*SADABS*; Krause *et al.*, 2015)	θ_max_ = 36.6°, θ_min_ = 2.8°
*T*_min_ = 0.595, *T*_max_ = 0.747	*h* = −19→19
18799 measured reflections	*k* = −23→23
3373 independent reflections	*l* = −14→14

### Pentacadmium tetrakis[oxidotellurate(IV)] dinitrate (Cd4Te5O14) Refinement

**Table:**

Refinement on *F*^2^	106 parameters
Least-squares matrix: full	0 restraints
*R*[*F*^2^ > 2σ(*F*^2^)] = 0.022	*w* = 1/[σ^2^(*F*_o_^2^) + (0.007*P*)^2^ + 7.0237*P*] where *P* = (*F*_o_^2^ + 2*F*_c_^2^)/3
*wR*(*F*^2^) = 0.040	(Δ/σ)_max_ = 0.001
*S* = 1.21	Δρ_max_ = 1.10 e Å^−^^3^
3373 reflections	Δρ_min_ = −1.52 e Å^−^^3^

### Pentacadmium tetrakis[oxidotellurate(IV)] dinitrate (Cd4Te5O14) Special details

**Table:**

Geometry. All esds (except the esd in the dihedral angle between two l.s. planes) are estimated using the full covariance matrix. The cell esds are taken into account individually in the estimation of esds in distances, angles and torsion angles; correlations between esds in cell parameters are only used when they are defined by crystal symmetry. An approximate (isotropic) treatment of cell esds is used for estimating esds involving l.s. planes.

### Pentacadmium tetrakis[oxidotellurate(IV)] dinitrate (Cd4Te5O14) Fractional atomic coordinates and isotropic or equivalent isotropic displacement parameters (Å^2^)

**Table:**

	*x*	*y*	*z*	*U*_iso_*/*U*_eq_	
Cd1	0.32186 (2)	0.14608 (2)	0.44068 (2)	0.01114 (4)	
Cd2	0.000000	0.15438 (2)	0.250000	0.01217 (5)	
Cd3	0.000000	0.52705 (2)	0.250000	0.01663 (6)	
Te1	0.15063 (2)	0.02884 (2)	0.04951 (2)	0.00946 (4)	
Te2	0.15858 (2)	0.33985 (2)	0.15582 (2)	0.01183 (4)	
Te3	0.000000	0.76224 (2)	0.250000	0.01223 (5)	
O1	0.0182 (2)	0.41329 (16)	0.0722 (3)	0.0174 (4)	
O2	0.0618 (2)	0.08242 (15)	0.5194 (3)	0.0153 (4)	
O3	0.0700 (2)	0.23908 (16)	0.0113 (3)	0.0174 (4)	
O4	0.15288 (19)	0.26766 (14)	0.3340 (2)	0.0111 (3)	
O5	0.16381 (18)	0.05898 (15)	0.2662 (2)	0.0115 (4)	
O6	0.19537 (19)	0.46166 (16)	0.3662 (3)	0.0141 (4)	
O7	0.3814 (2)	0.17177 (19)	0.2316 (3)	0.0214 (5)	

### Pentacadmium tetrakis[oxidotellurate(IV)] dinitrate (Cd4Te5O14) Atomic displacement parameters (Å^2^)

**Table:**

	*U*^11^	*U*^22^	*U*^33^	*U*^12^	*U*^13^	*U*^23^
Cd1	0.01052 (8)	0.01416 (9)	0.00843 (8)	−0.00111 (7)	0.00347 (6)	−0.00161 (6)
Cd2	0.00833 (11)	0.01007 (12)	0.01683 (13)	0.000	0.00370 (10)	0.000
Cd3	0.01015 (12)	0.01016 (12)	0.02670 (16)	0.000	0.00435 (11)	0.000
Te1	0.00891 (7)	0.00993 (7)	0.00892 (7)	−0.00007 (6)	0.00293 (5)	−0.00049 (6)
Te2	0.00967 (7)	0.01489 (8)	0.01080 (7)	0.00058 (6)	0.00397 (6)	0.00439 (6)
Te3	0.01272 (11)	0.00968 (10)	0.01572 (11)	0.000	0.00719 (9)	0.000
O1	0.0112 (9)	0.0151 (10)	0.0205 (10)	0.0015 (8)	0.0005 (8)	0.0000 (8)
O2	0.0174 (10)	0.0133 (9)	0.0168 (10)	0.0057 (8)	0.0084 (8)	0.0045 (8)
O3	0.0243 (11)	0.0148 (10)	0.0095 (9)	−0.0062 (9)	0.0030 (8)	−0.0013 (8)
O4	0.0142 (9)	0.0105 (8)	0.0085 (8)	−0.0014 (7)	0.0043 (7)	0.0010 (7)
O5	0.0112 (8)	0.0130 (9)	0.0097 (8)	−0.0010 (7)	0.0035 (7)	−0.0027 (7)
O6	0.0100 (8)	0.0163 (10)	0.0137 (9)	−0.0035 (7)	0.0024 (7)	0.0018 (8)
O7	0.0193 (11)	0.0301 (13)	0.0147 (10)	−0.0132 (10)	0.0070 (9)	−0.0012 (9)

### Pentacadmium tetrakis[oxidotellurate(IV)] dinitrate (Cd4Te5O14) Geometric parameters (Å, º)

**Table:**

Cd1—O7	2.235 (2)	Cd3—O6	2.330 (2)
Cd1—O4^i^	2.237 (2)	Cd3—O7^iv^	2.478 (3)
Cd1—O5	2.262 (2)	Cd3—O7^v^	2.478 (3)
Cd1—O1^ii^	2.314 (2)	Cd3—O1^vi^	2.860 (2)
Cd1—O6^i^	2.353 (2)	Cd3—O1^vii^	2.860 (2)
Cd1—O4	2.539 (2)	Te1—O2^viii^	1.873 (2)
Cd2—O4^iii^	2.327 (2)	Te1—O5	1.882 (2)
Cd2—O4	2.327 (2)	Te1—O6^ix^	1.936 (2)
Cd2—O5^iii^	2.339 (2)	Te1—O2^iii^	2.476 (2)
Cd2—O5	2.339 (2)	Te2—O1	1.859 (2)
Cd2—O2^iii^	2.395 (2)	Te2—O4	1.890 (2)
Cd2—O2	2.395 (2)	Te2—O3	1.928 (2)
Cd2—O3	2.809 (2)	Te2—O6	2.441 (2)
Cd2—O3^iii^	2.809 (2)	Te3—O7^v^	1.876 (2)
Cd3—O1^iii^	2.318 (2)	Te3—O7^iv^	1.876 (2)
Cd3—O1	2.318 (2)	Te3—O3^vi^	2.088 (2)
Cd3—O6^iii^	2.330 (2)	Te3—O3^vii^	2.088 (2)
			
O7—Cd1—O5	89.60 (8)	O2^viii^—Te1—O5	98.70 (9)
O4^i^—Cd1—O5	132.70 (7)	O2^viii^—Te1—O6^ix^	91.48 (10)
O7—Cd1—O1^ii^	82.92 (9)	O5—Te1—O6^ix^	92.74 (9)
O4^i^—Cd1—O1^ii^	90.83 (8)	O2^viii^—Te1—O2^iii^	76.42 (10)
O5—Cd1—O1^ii^	122.23 (8)	O5—Te1—O2^iii^	80.70 (8)
O7—Cd1—O6^i^	147.00 (9)	O6^ix^—Te1—O2^iii^	165.07 (9)
O4^i^—Cd1—O6^i^	75.74 (7)	O1—Te2—O4	108.01 (10)
O5—Cd1—O6^i^	80.31 (7)	O1—Te2—O3	89.85 (10)
O1^ii^—Cd1—O6^i^	76.40 (8)	O4—Te2—O3	86.37 (9)
O7—Cd1—O4	92.99 (8)	O1—Te2—O6	75.66 (9)
O4^i^—Cd1—O4	75.55 (8)	O4—Te2—O6	80.14 (8)
O5—Cd1—O4	78.97 (7)	O3—Te2—O6	155.89 (9)
O1^ii^—Cd1—O4	158.20 (7)	O7^v^—Te3—O7^iv^	92.57 (17)
O6^i^—Cd1—O4	115.38 (7)	O7^v^—Te3—O3^vi^	92.56 (10)
O4^iii^—Cd2—O4	91.55 (10)	O7^iv^—Te3—O3^vi^	86.72 (9)
O4^iii^—Cd2—O5^iii^	81.98 (7)	O7^v^—Te3—O3^vii^	86.72 (9)
O4—Cd2—O5^iii^	162.69 (7)	O7^iv^—Te3—O3^vii^	92.56 (10)
O4^iii^—Cd2—O5	162.69 (7)	O3^vi^—Te3—O3^vii^	178.97 (13)
O4—Cd2—O5	81.98 (7)	O7—Cd1—O4^i^	130.55 (8)
O5^iii^—Cd2—O5	108.48 (10)	Te2—O1—Cd1^x^	123.87 (11)
O4^iii^—Cd2—O2^iii^	95.61 (7)	Te2—O1—Cd3	116.49 (11)
O4—Cd2—O2^iii^	120.19 (7)	Cd1^x^—O1—Cd3	104.08 (9)
O5^iii^—Cd2—O2^iii^	76.60 (7)	Te1^xi^—O2—Cd2	115.99 (10)
O5—Cd2—O2^iii^	74.25 (7)	Te1^xi^—O2—Te1^iii^	103.58 (10)
O4^iii^—Cd2—O2	120.19 (7)	Cd2—O2—Te1^iii^	90.75 (7)
O4—Cd2—O2	95.61 (7)	Te2—O3—Te3^vii^	126.44 (11)
O5^iii^—Cd2—O2	74.24 (7)	Te2—O4—Cd1^i^	112.50 (10)
O5—Cd2—O2	76.60 (7)	Te2—O4—Cd2	113.86 (9)
O2^iii^—Cd2—O2	128.99 (11)	Cd1^i^—O4—Cd2	118.33 (9)
O1^iii^—Cd3—O1	90.65 (12)	Te2—O4—Cd1	113.10 (9)
O1^iii^—Cd3—O6^iii^	70.32 (8)	Cd1^i^—O4—Cd1	104.45 (8)
O1—Cd3—O6^iii^	76.77 (8)	Cd2—O4—Cd1	92.39 (7)
O1^iii^—Cd3—O6	76.77 (8)	Te1—O5—Cd1	120.92 (10)
O1—Cd3—O6	70.32 (8)	Te1—O5—Cd2	109.97 (9)
O6^iii^—Cd3—O6	132.57 (11)	Cd1—O5—Cd2	99.61 (8)
O1^iii^—Cd3—O7^iv^	138.48 (8)	Te1^iv^—O6—Cd3	126.44 (11)
O1—Cd3—O7^iv^	115.36 (8)	Te1^iv^—O6—Cd1^i^	113.29 (9)
O6^iii^—Cd3—O7^iv^	143.97 (8)	Cd3—O6—Cd1^i^	102.51 (8)
O6—Cd3—O7^iv^	82.22 (8)	Te1^iv^—O6—Te2	119.89 (10)
O1^iii^—Cd3—O7^v^	115.35 (8)	Cd3—O6—Te2	96.53 (7)
O1—Cd3—O7^v^	138.48 (8)	Cd1^i^—O6—Te2	91.58 (8)
O6^iii^—Cd3—O7^v^	82.22 (8)	Te3^xii^—O7—Cd1	121.22 (12)
O6—Cd3—O7^v^	143.97 (8)	Te3^xii^—O7—Cd3^xii^	100.54 (11)
O7^iv^—Cd3—O7^v^	66.35 (10)	Cd1—O7—Cd3^xii^	99.69 (10)

Symmetry codes: (i) −*x*+1/2, −*y*+1/2, −*z*+1; (ii) *x*+1/2, −*y*+1/2, *z*+1/2; (iii) −*x*, *y*, −*z*+1/2; (iv) −*x*+1/2, *y*+1/2, −*z*+1/2; (v) *x*−1/2, *y*+1/2, *z*; (vi) *x*, −*y*+1, *z*+1/2; (vii) −*x*, −*y*+1, −*z*; (viii) *x*, −*y*, *z*−1/2; (ix) −*x*+1/2, *y*−1/2, −*z*+1/2; (x) *x*−1/2, −*y*+1/2, *z*−1/2; (xi) *x*, −*y*, *z*+1/2; (xii) *x*+1/2, *y*−1/2, *z*.

### Tetracadmium pentaoxidotellurate(IV) (Cd5TeO34NO32) Crystal data

**Table:**

Cd_5_(TeO_3_)_4_(NO_3_)_2_	*F*(000) = 1212
*M**_r_* = 1388.42	*D*_x_ = 5.094 Mg m^−^^3^
Monoclinic, *P*2_1_/*c*	Mo *K*α radiation, λ = 0.71073 Å
*a* = 9.9442 (4) Å	Cell parameters from 3799 reflections
*b* = 5.6173 (2) Å	θ = 2.5–36.2°
*c* = 16.6136 (7) Å	µ = 12.19 mm^−^^1^
β = 102.737 (1)°	*T* = 300 K
*V* = 905.19 (6) Å^3^	Plate, colorless
*Z* = 2	0.08 × 0.03 × 0.02 mm

### Tetracadmium pentaoxidotellurate(IV) (Cd5TeO34NO32) Data collection

**Table:**

Bruker APEXII CCD diffractometer	3381 reflections with *I* > 2σ(*I*)
Radiation source: fine-focus sealed tube	*R*_int_ = 0.050
ω– and φ–scan	θ_max_ = 36.3°, θ_min_ = 2.9°
Absorption correction: multi-scan (*SADABS*; Krause *et al.*, 2015)	*h* = −16→16
*T*_min_ = 0.517, *T*_max_ = 0.747	*k* = −9→9
17368 measured reflections	*l* = −27→27
4388 independent reflections	

### Tetracadmium pentaoxidotellurate(IV) (Cd5TeO34NO32) Refinement

**Table:**

Refinement on *F*^2^	133 parameters
Least-squares matrix: full	0 restraints
*R*[*F*^2^ > 2σ(*F*^2^)] = 0.030	*w* = 1/[σ^2^(*F*_o_^2^) + (0.0232*P*)^2^] where *P* = (*F*_o_^2^ + 2*F*_c_^2^)/3
*wR*(*F*^2^) = 0.061	(Δ/σ)_max_ = 0.001
*S* = 0.99	Δρ_max_ = 1.56 e Å^−^^3^
4388 reflections	Δρ_min_ = −2.37 e Å^−^^3^

### Tetracadmium pentaoxidotellurate(IV) (Cd5TeO34NO32) Special details

**Table:**

Geometry. All esds (except the esd in the dihedral angle between two l.s. planes) are estimated using the full covariance matrix. The cell esds are taken into account individually in the estimation of esds in distances, angles and torsion angles; correlations between esds in cell parameters are only used when they are defined by crystal symmetry. An approximate (isotropic) treatment of cell esds is used for estimating esds involving l.s. planes.

### Tetracadmium pentaoxidotellurate(IV) (Cd5TeO34NO32) Fractional atomic coordinates and isotropic or equivalent isotropic displacement parameters (Å^2^)

**Table:**

	*x*	*y*	*z*	*U*_iso_*/*U*_eq_	
Cd1	0.00202 (3)	0.42718 (5)	0.15555 (2)	0.01465 (6)	
Cd2	0.21172 (3)	0.44982 (4)	0.36945 (2)	0.01363 (6)	
Cd3	0.000000	0.000000	0.000000	0.01447 (8)	
Te1	0.25779 (3)	0.52156 (4)	0.01753 (2)	0.01033 (5)	
Te2	0.78086 (3)	0.41573 (4)	0.28168 (2)	0.01047 (5)	
N1	0.4949 (5)	0.0392 (8)	0.0997 (2)	0.0293 (9)	
O1	0.0761 (3)	0.0414 (5)	0.13841 (16)	0.0160 (6)	
O2	0.1195 (3)	0.6723 (5)	0.25746 (15)	0.0127 (5)	
O3	0.1359 (3)	0.2104 (4)	0.46235 (16)	0.0137 (5)	
O4	0.1855 (3)	0.1145 (5)	0.30190 (18)	0.0203 (6)	
O5	0.1876 (3)	0.5679 (5)	0.11038 (17)	0.0182 (6)	
O6	0.3682 (4)	0.0604 (8)	0.0832 (3)	0.0434 (10)	
O7	0.4500 (5)	0.3493 (8)	0.4139 (3)	0.0530 (12)	
O8	0.5720 (5)	0.2089 (9)	0.1271 (3)	0.0551 (12)	
O9	0.8429 (3)	0.2369 (4)	0.04836 (16)	0.0138 (5)	

### Tetracadmium pentaoxidotellurate(IV) (Cd5TeO34NO32) Atomic displacement parameters (Å^2^)

**Table:**

	*U*^11^	*U*^22^	*U*^33^	*U*^12^	*U*^13^	*U*^23^
Cd1	0.01571 (14)	0.01295 (11)	0.01570 (12)	−0.00266 (10)	0.00435 (10)	−0.00525 (9)
Cd2	0.02103 (15)	0.00881 (10)	0.01172 (11)	0.00022 (10)	0.00505 (10)	0.00000 (8)
Cd3	0.0210 (2)	0.01277 (15)	0.01182 (16)	−0.00376 (15)	0.00839 (15)	−0.00284 (13)
Te1	0.01106 (11)	0.00940 (9)	0.01050 (10)	0.00022 (8)	0.00232 (8)	−0.00003 (7)
Te2	0.01209 (12)	0.00921 (9)	0.01048 (10)	0.00040 (8)	0.00330 (8)	−0.00012 (7)
N1	0.022 (2)	0.042 (2)	0.0236 (19)	−0.0042 (19)	0.0051 (16)	0.0085 (17)
O1	0.0220 (16)	0.0136 (12)	0.0116 (12)	0.0061 (11)	0.0021 (11)	0.0020 (9)
O2	0.0152 (14)	0.0128 (11)	0.0100 (11)	−0.0037 (10)	0.0027 (10)	0.0012 (9)
O3	0.0166 (15)	0.0095 (11)	0.0152 (12)	0.0043 (10)	0.0041 (11)	0.0038 (9)
O4	0.0251 (17)	0.0159 (13)	0.0214 (14)	−0.0027 (12)	0.0088 (12)	−0.0089 (11)
O5	0.0222 (16)	0.0200 (13)	0.0141 (12)	−0.0042 (12)	0.0076 (12)	−0.0041 (10)
O6	0.0172 (19)	0.059 (3)	0.054 (2)	0.0039 (18)	0.0081 (17)	0.024 (2)
O7	0.041 (3)	0.039 (2)	0.075 (3)	−0.010 (2)	0.003 (2)	−0.001 (2)
O8	0.036 (3)	0.065 (3)	0.058 (3)	−0.014 (2)	−0.003 (2)	−0.016 (2)
O9	0.0165 (15)	0.0084 (11)	0.0158 (12)	0.0016 (10)	0.0023 (11)	0.0006 (9)

### Tetracadmium pentaoxidotellurate(IV) (Cd5TeO34NO32) Geometric parameters (Å, º)

**Table:**

Cd1—O5	2.281 (3)	Cd3—O1^v^	2.269 (3)
Cd1—O2	2.292 (3)	Cd3—O3^vi^	2.289 (3)
Cd1—O1	2.326 (3)	Cd3—O3^iii^	2.289 (3)
Cd1—O9^i^	2.361 (3)	Cd3—O9^vii^	2.326 (3)
Cd1—O4^ii^	2.380 (3)	Cd3—O9^i^	2.326 (3)
Cd1—O2^iii^	2.524 (3)	Te1—O5	1.847 (3)
Cd1—O3^ii^	2.658 (3)	Te1—O3^vi^	1.875 (3)
Cd2—O4	2.179 (3)	Te1—O9^viii^	1.886 (3)
Cd2—O9^iv^	2.256 (3)	Te2—O1^iv^	1.858 (3)
Cd2—O2	2.261 (3)	Te2—O4^iv^	1.869 (3)
Cd2—O3	2.296 (3)	Te2—O2^ix^	1.886 (3)
Cd2—O7	2.389 (5)	N1—O6	1.234 (5)
Cd2—O8^iv^	2.587 (5)	N1—O7^ix^	1.242 (6)
Cd3—O1	2.269 (3)	N1—O8	1.245 (6)
			
O5—Cd1—O2	73.58 (9)	O3^iii^—Cd3—O9^vii^	99.77 (9)
O5—Cd1—O1	88.94 (10)	O1—Cd3—O9^i^	71.97 (10)
O2—Cd1—O1	121.76 (10)	O1^v^—Cd3—O9^i^	108.03 (10)
O5—Cd1—O9^i^	111.40 (10)	O3^vi^—Cd3—O9^i^	99.77 (9)
O2—Cd1—O9^i^	167.59 (10)	O3^iii^—Cd3—O9^i^	80.23 (9)
O1—Cd1—O9^i^	70.34 (10)	O9^vii^—Cd3—O9^i^	180.00 (16)
O5—Cd1—O4^ii^	133.48 (10)	O5—Te1—O3^vi^	100.59 (12)
O2—Cd1—O4^ii^	79.64 (10)	O5—Te1—O9^viii^	97.66 (12)
O1—Cd1—O4^ii^	137.57 (10)	O3^vi^—Te1—O9^viii^	90.73 (12)
O9^i^—Cd1—O4^ii^	89.17 (10)	O1^iv^—Te2—O4^iv^	93.99 (13)
O5—Cd1—O2^iii^	155.39 (10)	O1^iv^—Te2—O2^ix^	98.29 (12)
O2—Cd1—O2^iii^	98.53 (6)	O4^iv^—Te2—O2^ix^	89.04 (12)
O1—Cd1—O2^iii^	75.29 (9)	Te2^ix^—O1—Cd3	135.81 (14)
O9^i^—Cd1—O2^iii^	81.33 (9)	Te2^ix^—O1—Cd1	118.66 (13)
O4^ii^—Cd1—O2^iii^	64.87 (9)	Cd3—O1—Cd1	100.13 (10)
O4—Cd2—O9^iv^	155.85 (11)	Te2^iv^—O2—Cd2	122.40 (13)
O4—Cd2—O2	94.20 (10)	Te2^iv^—O2—Cd1	113.69 (11)
O9^iv^—Cd2—O2	89.70 (9)	Cd2—O2—Cd1	108.91 (11)
O4—Cd2—O3	79.63 (10)	Te2^iv^—O2—Cd1^ii^	98.06 (11)
O9^iv^—Cd2—O3	81.57 (9)	Cd2—O2—Cd1^ii^	90.05 (8)
O2—Cd2—O3	138.01 (10)	Cd1—O2—Cd1^ii^	122.22 (12)
O4—Cd2—O7	87.31 (14)	Te1^x^—O3—Cd3^ii^	135.82 (13)
O9^iv^—Cd2—O7	109.67 (13)	Te1^x^—O3—Cd2	117.59 (13)
O2—Cd2—O7	125.43 (13)	Cd3^ii^—O3—Cd2	93.93 (9)
O3—Cd2—O7	95.97 (13)	Te2^ix^—O4—Cd2	151.47 (17)
O4—Cd2—O8^iv^	120.16 (14)	Te2^ix^—O4—Cd1^iii^	103.71 (13)
O9^iv^—Cd2—O8^iv^	83.95 (13)	Cd2—O4—Cd1^iii^	104.00 (11)
O2—Cd2—O8^iv^	83.78 (12)	Te1—O5—Cd1	135.10 (15)
O3—Cd2—O8^iv^	135.16 (12)	N1^iv^—O7—Cd2	100.8 (3)
O7—Cd2—O8^iv^	50.43 (14)	N1—O8—Cd2^ix^	91.1 (3)
O1—Cd3—O1^v^	180.0	Te1^viii^—O9—Cd2^ix^	134.23 (15)
O1—Cd3—O3^vi^	96.84 (10)	Te1^viii^—O9—Cd3^xi^	121.47 (12)
O1^v^—Cd3—O3^vi^	83.16 (10)	Cd2^ix^—O9—Cd3^xi^	94.00 (9)
O1—Cd3—O3^iii^	83.16 (10)	Te1^viii^—O9—Cd1^xi^	107.10 (11)
O1^v^—Cd3—O3^iii^	96.84 (10)	Cd2^ix^—O9—Cd1^xi^	94.47 (9)
O3^vi^—Cd3—O3^iii^	180.00 (15)	Cd3^xi^—O9—Cd1^xi^	97.48 (10)
O1—Cd3—O9^vii^	108.03 (10)	O6—N1—O7^ix^	120.8 (5)
O1^v^—Cd3—O9^vii^	71.97 (10)	O6—N1—O8	121.6 (5)
O3^vi^—Cd3—O9^vii^	80.23 (9)	O7^ix^—N1—O8	117.6 (5)

Symmetry codes: (i) *x*−1, *y*, *z*; (ii) −*x*, *y*+1/2, −*z*+1/2; (iii) −*x*, *y*−1/2, −*z*+1/2; (iv) −*x*+1, *y*+1/2, −*z*+1/2; (v) −*x*, −*y*, −*z*; (vi) *x*, −*y*+1/2, *z*−1/2; (vii) −*x*+1, −*y*, −*z*; (viii) −*x*+1, −*y*+1, −*z*; (ix) −*x*+1, *y*−1/2, −*z*+1/2; (x) *x*, −*y*+1/2, *z*+1/2; (xi) *x*+1, *y*, *z*.
